# Plasma lipid profile associates with the improvement of psychological well-being in individuals with perceived stress symptoms

**DOI:** 10.1038/s41598-020-59051-x

**Published:** 2020-02-07

**Authors:** Stefania Noerman, Anton Klåvus, Elina Järvelä-Reijonen, Leila Karhunen, Seppo Auriola, Riitta Korpela, Raimo Lappalainen, Urho M. Kujala, Sampsa Puttonen, Marjukka Kolehmainen, Kati Hanhineva

**Affiliations:** 1 Institute of Public Health and Clinical Nutrition, University of Eastern Finland, P.O. Box 1627, FI-70211 Kuopio, Finland; 2 School of Pharmacy, University of Eastern Finland, Kuopio, Finland; 3 LC-MS Metabolomics Centre, Biocentre Kuopio, Kuopio, Finland; 4 Medical Faculty, Pharmacology and Human Microbe Research program, University of Helsinki, P.O. Box 63, FI-00014 Helsinki, Finland; 5 Department of Psychology, Faculty of Education and Psychology, University of Jyväskylä, PO Box 35, FI-40014 Jyväskylä, Finland; 6 Faculty of Sport and Health Sciences, University of Jyväskylä, P.O. Box 35, FI-40014 Jyväskylä, Finland; 7 Finnish Institute of Occupational Health, P.O. Box 40, FI-00251 Helsinki, Finland

**Keywords:** Metabolomics, Predictive markers

## Abstract

Psychological stress is a suggested risk factor of metabolic disorders, but molecular mediators are not well understood. We investigated the association between the metabolic profiles of fasting plasma and the improvement of psychological well-being using non-targeted liquid chromatography-mass spectrometry (LC-MS) platform. The metabolic profiles of volunteers participating in the face-to-face intervention group (n = 60) in a randomised lifestyle intervention were compared to ones of controls (n = 64) between baseline and 36-week follow-up. Despite modest differences in metabolic profile between groups, we found associations between phosphatidylcholines (PCs) and several parameters indicating stress, adiposity, relaxation, and recovery. The relief of heart-rate-variability-based stress had positive, while improved indices of recovery and relaxation in the intervention group had an inverse association with the reduction of *e.g*. lysophosphatidylcholines (LPC). Interleukin-1 receptor antagonist and adiposity correlated positively with the suppressed PCs and negatively with the elevated plasmalogens PC(P-18:0/22:6) and PC(P-18:0/20:4). Also, we found changes in an unknown class of lipids over time regardless of the intervention groups, which also correlated with physiological and psychological markers of stress. The associations between lipid changes with some markers of psychological wellbeing and body composition may suggest the involvement of these lipids in the shared mechanisms between psychological and metabolic health.

## Introduction

The co-existence of psychological stress with higher adiposity^1^ and obesity^2^ has nominated stress as an essential determinant of health. In a physiological context, stress has been associated with hypertension^3^, alteration of blood glucose and lipid profiles^4^, elevated blood pressure, heart rate, and fibrinogen responses^5^, suggesting stress as a risk factor of metabolic disorders^6,7^. The biological mechanisms linking psychological stress with atherosclerosis and obesity^8^ have been previously proposed, including inflammation and altered activity of the hypothalamic-pituitary-adrenal (HPA) axis or neuroendocrine pathway^1^.

In addition to the biological implications, psychological stress has also been linked to detrimental health-related habits or lifestyle leading to health deterioration, including increased smoking^9^ and alcohol intake^2^. Furthermore, stress has been accompanied by unfavourable eating behaviours, such as emotional^10^, uncontrolled eating, and higher frequency of binge eating^11^. Stress has also been associated with a poor dietary quality^1^, reflected by higher intake of fast-food and calorie derived from saturated fat and sugar^2,11–13^, and lower consumption of fruits, vegetables, legumes, and whole grains^11^. Thus, the contribution of psychological stress to obesity or other metabolic disturbances cannot rule out behavioural changes. This complex interaction hence provides a challenge^8^ to fully comprehend the causal relationship between psychological well-being and metabolic health in a perspective of the human body as a system. Therefore, there is a need to clarify this relationship to enable more effective prevention of both obesity and psychological stress.

One of the ways to elucidate the relationship is by assessing the implication of psychological stress in the systemic circulation. Several experimental studies have previously shown how the profile of blood metabolites could reflect behavioural symptoms. In mice, stress has been indicated by higher plasma levels of 4-hydroxyproline, 3-aminopropanoic acid, orotic acid, and lower urea, dihydroxyacetone, ribose, lactic acid, and glycerol^14^. In dogs, behavioural scores similar to attention-deficit/hyperactivity disorder have been associated positively with kynurenic acid, fatty acids (20:4) and (18:1), and negatively with phospholipids and microbial metabolites of tryptophan such as indole acetic acid and 3-indolepropionic acid^15^. In humans, similarly, kynurenine and indoxyl sulfate have been associated with a personality trait prone to psychological stress^16^. Depression has been marked by a lower plasma level of medium-chain fatty acids^17^ and the ratio between fatty acids (20:4) and (22:5), independent of dietary consumption of n-3 or n-6 fatty acids^18^.

**Figure 1:**
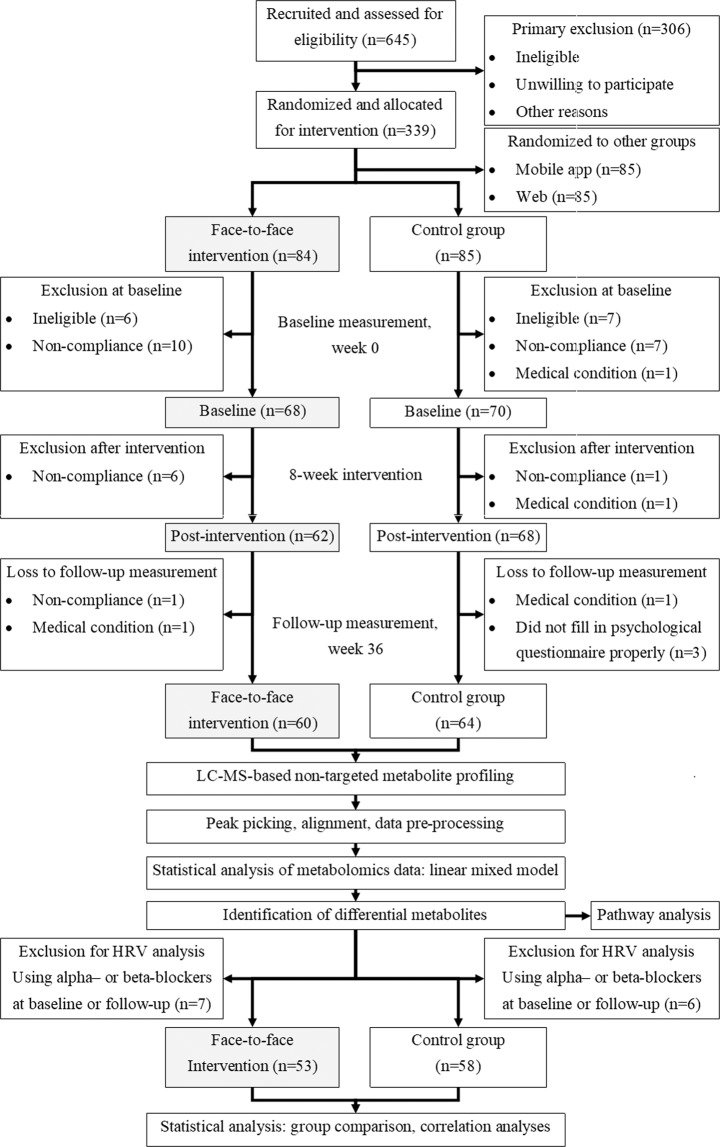
Working flowchart of metabolomics approach on sample collection of Elixir study. HRV: heart-rate variability.

Elixir study was a multi-centre lifestyle intervention aimed to enhance the psychological well-being of 339 individuals with overweight/obesity and self-reported stress symptoms^19^. In this study, higher perceived stress was associated with less physical activity^20^ and unfavourable eating behaviours, such as higher dependency on emotional and rewarding properties of food rather than physiological hunger cues and less consumption of wholegrain products^21^. Conversely, study participants with higher physical activity recovered better from stress^22^. Considering these results, we aimed to examine whether the improvement of psychological well-being in the volunteers of the Elixir study is associated with specific profiles of circulating metabolites that would indicate both subjective and objective measures of psychological well-being. We investigated the metabolic profiles in the volunteers of the control, as well as of the face-to-face intervention group (Fig. 1) representing the group with the most noticeable improvement of their psychological stress levels among other intervention groups^23^. In this report, we show how specific plasma metabolites reflect changes in psychological well-being and body composition, which may assist further studies aiming to elucidate the causal mechanisms linking psychological well-being and metabolic health.

## Results

### Body composition and lifestyle indicators

Within this subset of the 8-week lifestyle intervention study, the participants in the intervention (n = 60) and control groups (n = 64) had a similar distribution of gender, seasons of enrollment, and research centres (Table 1). The body composition and distribution of lifestyle features (alcohol consumption, smoking, and physical activity) at baseline were similar, too (Table 1).

**Table 1:** Changes of the participants’ body composition and lifestyle indicators.

Study groups	Baseline (week 0)			After follow-up (week 36)			Changes (week 36 - week 0)					
	Intervention	Control	p	Intervention	Control	p	Intervention	p	Control	p	p	Cohen’s
	(n = 60)	(n = 64)	(I0-C0)	(n = 60)	(n = 64)	(I36-C36)		(dI)		(dC)	(dI-dC)	d
Age (years)	50.7 ± 7.2	49.5 ± 7.4	0.282									
Gender (males/females)	6/54	11/53	0.245									
Season of enrollment (autumn/spring)	29/31	28/36	0.609									
Research centres (Jyväskylä/Kuopio/Helsinki)	16/19/25	15/21/28	0.917									
Users of alpha/beta-blockers	7	6										
Body composition												
Body weight (kg)	85.2 ± 10.2	88.0 ± 11.7	0.181	83.5 ± 10.7	87.1 ± 11.7	0.088	−1.6 ± 3.4	**0.001**	−0.9 ± 2.5	**0.003**	0.375	−0.257
BMI (kg/m^2^)	30.8 ± 3.0	31.1 ± 2.9	0.417	30.2 ± 3.2	30.8 ± 3.1	0.177	−0.6 ± 1.3	**0.001**	−0.3 ± 0.9	**0.004**	0.337	−0.280
Waist circumference (cm)	100.5 ± 8.7	103.5 ± 9.3	0.074	98.0 ± 10.2	101.8 ± 10.0	0.059	−2.5 ± 4.1	**<0.001**	−1.7 ± 3.2	**<0.001**	0.347	−0.219
Body fat (%)	39.2 ± 5.9	38.9 ± 6.5	0.869	38.1 ± 6.3	38.5 ± 6.6	0.587	−1.2 ± 2.1	**<0.001**	−0.4 ± 2.2	**0.017**	0.136	−0.331
Alcohol usage (% users)	58.3	65.6	0.237	41.7	60.9	**0.002**	−16.6	**<0.001**	−4.7	0.286	0.107	
AUDIT-C score^a^	3.2 ± 2.1	3.9 ± 2.4	0.134	2.7 ± 2.00	3.6 ± 2.3	**0.039**	−0.5 ± 1.1	**<0.001**	−0.3 ± 1.7	**0.026**	0.493	−0.112
Smoking (% smokers)			0.787			0.781		1.000		0.206		
Never smoked	58.3	53.1		60.0	56.3		1.7		3.2			
Regularly	6.7	9.4		5.0	7.8		−1.7		−1.6			
Randomly	6.7	6.3		5.0	6.3		−1.7		0			
Quit smoking	28.3	31.3		30.0	29.7		1.7		−1.6			
Physical activity												
LTPA (MET h/d)^b^	3.68 ± 2.94	2.92 ± 2.92	0.054	4.27 ± 3.26	2.86 ± 2.73	**0.004**	0.59 ± 3.48	0.521	−0.06 ± 2.20	0.412	0.912	0.230

Unless indicated otherwise, presented values are mean ± SD.

Cohen’s D indicates the differences between changes in the intervention and the control groups, divided by the mean of the standard deviation of the changes within both groups.

*P*-values within groups and between groups were tested using Wilcoxon signed-rank and Mann-Whitney U tests, respectively. Group proportion was compared using Chi-Square test for differences between groups. For differences within the group, McNemar and marginal homogeneity test were used for the proportion of alcohol and smoking users, respectively. P < 0.05 were considered significant (printed in bold).

^a^AUDIT-C: Alcohol Use Disorders Identification Test-Consumption.

^b^LTPA: Leisure-time physical activity, estimated using the metabolic equivalent (MET) hour per day.

After the follow-up (week 36), both intervention and control groups within this substudy showed changes in their body composition, marked by the reduction of body weight, BMI, waist circumference, and body fat percentage, but the changes did not differ between the groups (Table 1). At the follow-up, the alcohol consumption based on AUDIT-C questionnaire was reduced in both groups so that the changes between the groups were not significantly different (*p* between-group difference for changes = 0.493). The smoking habit did not change in either of the groups. Compared to the control group, the intervention group had more leisure-time physical activity (LTPA) at the follow-up (*p* between-group difference = 0.004), though the change in LTPA did not seem to reach statistical significance (*p* change within intervention group = 0.521) nor lead to between-group difference (*p* between-group difference for changes = 0.912). The effect sizes for the reduction of adiposity, alcohol consumption, and increased physical activity were considered medium (Cohen’s D < −0.25).

### Sleep, stress assessment, and inflammation markers

Because the medications containing alpha- and beta-blockers may potentially affect heart rate variability (HRV), 7 and 6 volunteers using such medicines (Table 1) were excluded in all analyses using objective HRV-based stress measurements from the intervention and control groups, respectively (Fig. 1). Therefore, the results with HRV-based parameters concerned 53 volunteers in the intervention and 58 volunteers in the control group.

Based on the subjective Perceived Stress Scale (PSS) questionnaire, the stress levels decreased in both groups, but the differences between the groups were not significant (*p* between-group difference for changes = 0.271) (Table 2). Psychological flexibility, as indicated by the inversely-scaled Acceptance and Action Questionnaire for Weight-related difficulties (AAQW), improved in both groups, and the reduction in the intervention group was higher than in the control group (*p* = 0.013), as also indicated by the large effect size (Cohen’s D = -0.554). The increased HRV-based index of stress in the control group (*p* = 0.037) indicated higher stress in the control group at the follow-up, making the changes significantly different from the intervention group (*p* = 0.014, Cohen’s D = -0.400). The HRV-based relaxation index increased more (*p* = 0.012) in the intervention group than in the control group (*p* for between-group changes = 0. 039, Cohen’s D = 0.162), along with a trend of elevated average Root Mean Square of the Successive R-R Differences (RMSSD) (*p* change within the intervention group = 0.033, *p* changes between groups = 0.052), showing tendency of better relaxation processes in the intervention group (Table 2). The sleep duration, efficiency, and quality did not change in the intervention group, though the control group seemed to improve their sleep efficiency (*p* changes within control group = 0.043) due to lower sleep efficiency at baseline (*p* differences between groups = 0.035). Highly sensitive C-reactive protein (hsCRP) was the only inflammation marker with a significant reduction within the intervention group (*p* = 0.023), but the between-group difference for the change did not reach statistical significance (*p* = 0.176) (Table 2).

**Table 2:** Changes of the participants’ indicators of psychological well-being, sleep parameters, and inflammation markers, excluding the users of alpha- and beta-blockers medication.

Study groups	Baseline (week 0)			After follow-up (week 36)			Changes (week 36 - week 0)					
	Intervention	Control	p	Intervention	Control	p	Intervention	p	Control	p	p	Cohen’s
	(n = 53)	(n = 58)	(I0-C0)	(n = 53)	(n = 58)	(I36-C36)		(dI)		(dC)	(dI-dC)	d
Subjective indicators of psychological well-being												
Perceived Stress Scale (PSS)	25.1 ± 7.9	26.1 ± 7.8	0.615	20.1 ± 8.1	22.9 ± 9.4	0.133	−5.0 ± 9.1	** < 0.001**	−3.2 ± 9.0	**0.007**	0.271	−0.220
AAQW^a^	84.1 ± 19.8	87.6 ± 21.3	0.285	72.0 ± 20.8	83.0 ± 23.8	**0.012**	−12.4 ± 14.9	** < 0.001**	−6.2 ± 13.0	**0.004**	**0.013**	−0.554
Objective indicators of psychological well-being												
Stress Balance during Sleep	0.43 ± 0.44	0.26 ± 0.45	**0.047**	0.39 ± 0.51	0.19 ± 0.50	**0.017**	−0.05 ± 0.64	0.935	−0.09 ± 0.62	0.305	0.443	0.054
Stress Index	167.7 ± 40.4	165.9 ± 67.2	0.320	166.3 ± 42.6	164.2 ± 51.9	0.531	−4.1 ± 19.3	0.134	5.8 ± 29.9	**0.037**	**0.014**	−0.400
Relaxation Index	80.2 ± 7.9	82.6 ± 9.2	0.056	80.7 ± 7.9	83.0 ± 8.51	0.094	0.8 ± 5.4	**0.012**	0.1 ± 3.0	0.881	**0.039**	0.162
Recovery Index	69.1 ± 33.7	71.4 ± 27.2	0.366	70.0 ± 32.0	70.9 ± 27.4	0.654	2.1 ± 22.1	0.361	−2.5 ± 20.4	0.176	0.141	0.218
Relaxation percentage	26.5 ± 8.9	23.1 ± 8.9	**0.031**	26.5 ± 12.0	22.6 ± 11.1	0.103	0.6 ± 15.0	0.762	−0.7 ± 13.1	0.386	0.389	0.092
Stress Percentage	47.5 ± 10.0	50.2 ± 10.9	0.295	50.8 ± 11.9	51.9 ± 13.6	0.781	2.7 ± 14.0	0.096	1.5 ± 12.8	0.315	0.657	0.088
Average RMSSD^b^	25.0 ± 11.5	26.9 ± 11.1	0.167	25.4 ± 9.2	27.6 ± 12.4	0.387	1.5 ± 5.8	**0.033**	0.4 ± 8.1	0.515	0.052	0.160
Subjective sleep-related parameters												
Sleep duration (hours)	6.06 ± 1.03	5.74 ± 1.17	0.189	5.90 ± 1.21	6.09 ± 1.16	0.523	−0.18 ± 1.13	0.335	0.29 ± 1.10	0.143	0.099	−0.419
Sleep efficiency (%)^c^	80.1 ± 11.2	74.9 ± 13.0	**0.035**	80.2 ± 12.6	78.9 ± 11.7	0.270	−0.2 ± 13.4	0.838	3.5 ± 13.8	**0.043**	0.234	−0.273
Sleep quality^d^	3.20 ± 0.51	3.34 ± 0.49	0.213	3.39 ± 0.60	3.38 ± 0.58	0.973	0.20 ± 0.63	0.056	0.04 ± 0.52	0.501	0.312	0.294
Inflammation markers^e^												
HMW adiponectin (µg/ml)	5.77 ± 3.75	6.02 ± 5.79	0.480	6.10 ± 4.35	5.83 ± 4.76	0.516	0.33 ± 1.71	0.108	−0.19 ± 2.08	0.567	0.392	0.272
IL-1Ra (ng/ml)	0.40 ± 0.19	0.46 ± 0.21	0.111	0.38 ± 0.19	0.41 ± 0.17	0.107	−0.02 ± 0.14	0.298	−0.05 ± 0.17	0.140	0.719	0.189
hsCRP (mg/l)	3.66 ± 6.04	3.88 ± 5.85	0.320	2.30 ± 2.69	4.13 ± 7.33	0.098	−1.37 ± 4.98	**0.023**	−0.25 ± 8.43	0.952	0.176	−0.242

Unless indicated otherwise, presented values are mean ± SD.

Cohen’s D indicates the differences between changes in the intervention and the control groups, divided by the mean of the standard deviation of the changes within both groups.

P-values within groups and between groups were tested using Wilcoxon signed-rank and Mann-Whitney U tests, respectively. P < 0.05 were significant (printed in bold).

^a^AAQW: weight-related Acceptance and Action Questionnaire, a higher score indicates psychological inflexibility related to body weight and appearance.

^b^Average RMSSD: the average of root-mean-square of successive differences between beats within 5-minute intervals during sleeping time.

^c^Sleep efficiency: the ratio of total sleep duration to the total time spent in bed.

^d^Sleep quality was presented as the mean of 5-scale sleep quality (1 is the worst, 5 is the best) from day 1–7 since study visits.

^e^HMW adiponectin: high-molecular-weight adiponectin; IL-1Ra: interleukin-1-receptor antagonist; hsCRP: highly-sensitive C reactive protein.

### The correlation between metabolites and physiological and psychological parameters

The linear mixed model with the interaction between time and intervention as the main result did not give any statistically differential features after the Benjamini-Hochberg false discovery rate (FDR) based on the number of unannotated features (n = 2800) (Supplementary Fig. 1). However, our earlier analyses have shown that the intervention group had the most pronounced differences in psychological flexibility^23^, and hence we proceeded to correlate the nominally differential metabolites (unadjusted *p* < 0.05) to evaluate whether the participants’ metabolite profiles were associated with the changes in stress-related measurements conducted during the study. Among 104 nominally differential features with unadjusted *p* < 0.05, many of the metabolites were not identifiable due to low signal intensities, resulting in lack of MS/MS data. After removal of redundant features, we identified 27 metabolites, 17 of them belonged to the PC class (Supplementary Table S1**)**. Concurrent with the high prevalence of PC identified, glycerophospholipid metabolism topped the chart of pathway analysis with FDR 0.003 (Supplementary Table S2). The correlation analyses were hence performed on these 27 identified metabolites.

**Figure 2:**
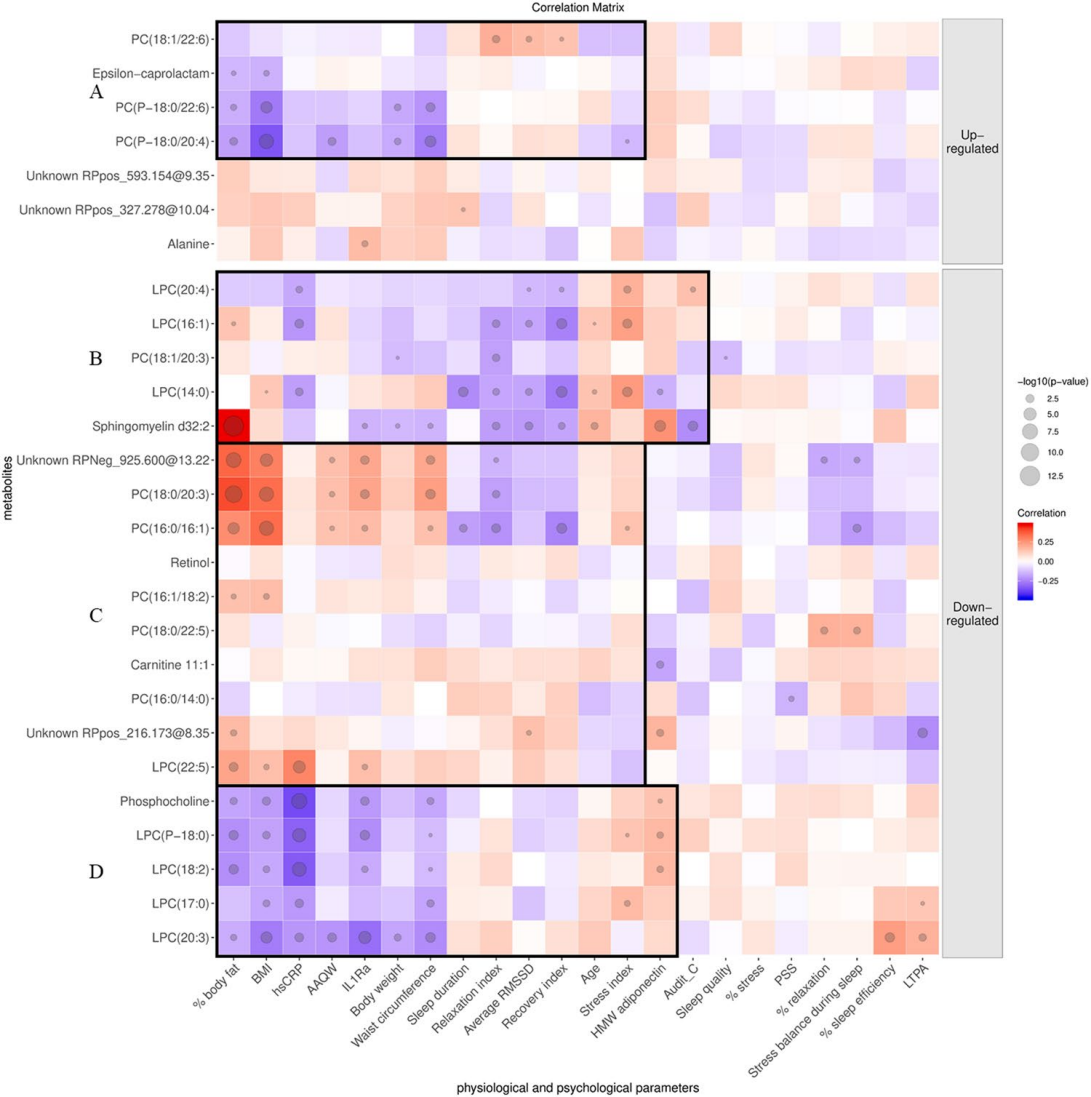
Correlation matrix between metabolites and the physiological and psychological parameters of the study participants. The up-regulated metabolites had a positive estimate, indicating an increase in the intervention group or a reduction in the control group, while the down-regulated ones had a negative estimate (reduced in the intervention group or elevated in the control group). Red blocks indicate a positive correlation, and blue blocks indicate otherwise. The size of the circle indicates –log p. AAQW: Acceptance and Action Questionnaire for Weight-related difficulties; AUDIT-C: Alcohol Use Disorders Identification Test-Consumption BMI: body mass index; HMW adiponectin: high-molecular-weight adiponectin; hsCRP: highly sensitive C-reactive protein; IL-1Ra: interleukin-1 receptor antagonist; (L)PC: (lyso)phosphatidylcholine; LTPA: leisure-time physical activity; PSS: Perceived Stress Scale; RMSSD: Root Mean Square of the Successive R-R Differences; RP: reversed-phase.

Along with the reduction of adiposity, including % body fat, body weight, BMI, waist circumference (Table 1), plasmalogens PC(P-18:0/22:6) and PC(P-18:0/20:4) increased in the intervention group, and correlated negatively with adiposity indicators (Cluster A, Fig. 2**)**. In particular, PC(P-18:0/20:4) also inversely correlated with HRV-based stress index and the inverse-scaled AAQW, which showed improvement of psychological flexibility. Another elevated PC in the intervention group, PC(18:1/22:6), correlated positively with average RMSSD, recovery and relaxation indices (Cluster A, Fig. 2), along with the improvement of relaxation index and average RMSSD in the intervention group (Table 2).

The improvement of stress indicators in the intervention group (Table 2) went along with the suppressed LPC(14:0), (16:1), and (20:4) since those LPCs correlated positively with stress and inversely with recovery indices and average RMSSD (Cluster B, Fig. 2). The improved indicators of adiposity (Table 1) and AAQW (Table 2) in the intervention group had a positive correlation with the suppression of PC(16:0/16:1), (18:0/20:3), and an unknown metabolite 925.600@13.22 at RP negative (Cluster C, Fig. 2). Also, these suppressed lipids correlated positively with inflammation marker interleukin-1 receptor antagonist (IL-1Ra) and negatively with relaxation index. Within the same cluster, the suppressed LPC(22:5) had a positive correlation with BMI, body fat, IL-1Ra, and hsCRP **(**Fig. 2).

Phosphocholine and other down-regulated LPCs were associated positively with HMW adiponectin and inversely with indicators of inflammation and body composition (Cluster D, Fig. 2). Because these down-regulated metabolites may be attributable to the increased levels in the control group, this correlation was most likely related to the improvement in the control group (Table 1).

### The metabolites that changed similarly in both intervention and control groups

The *p* distribution for the effect of time was skewed towards small *p*-values compared to the uniform distribution of random p-values expected under the null hypothesis (Supplementary Fig. S1). 36 features had FDR < 0.05, which were refined to 13 features with RT >1 min. They increased in both groups by time and were lipids, except for one metabolite eluting at RT 3.93 min in Hilic. Among them, only five features can be annotated based on the MS/MS spectra. One metabolite was LPC(P-18:0) which also had *p* < 0.05 for interaction between time and intervention, and one was a PC dimer that eluted early in the Hilic so that it was discarded due to low reliability of MS/MS data for identification purpose. Noteworthy, 9 of these 12 lipids without any hit from the database search for the mass, eluted at 5–6 mins in RP column, which was quite unusual for such large molecules. Based on the available MS/MS spectra of two of those, these molecules had neutral losses of 17 from the precursor ion and neutral losses of 44 from the product ions. Also, the similar fragments with *m/z* 133.085 were observed in both features. 7 out of 9 lipids were doubly-charged, which may indicate another polar head for this class of lipid, e.g. glyco- or aminolipids. However, due to lack of features’ MS/MS spectra and reference spectral library, the annotation could not continue.

**Figure 3:**
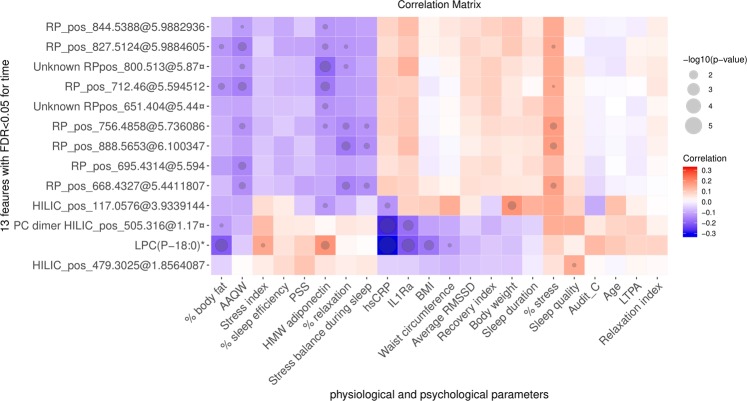
The correlation analysis between physiological and psychological parameters with 13 features differential for time (FDR < 0.05) which increased in both intervention and control groups. *Indicates metabolite that also differed for interaction between time and intervention, and ¤ indicate metabolites which have been annotated based on the MS/MS spectra. Red blocks indicate a positive correlation, and blue blocks indicate otherwise. The size of the circle indicates –log p. AAQW: Acceptance and Action Questionnaire for Weight-related difficulties; AUDIT-C: Alcohol Use Disorders Identification Test-Consumption BMI: body mass index; HILIC: hydrophilic interaction column; HMW adiponectin: high-molecular-weight adiponectin; hsCRP: highly sensitive C-reactive protein; IL-1Ra: interleukin-1 receptor antagonist; LPC: (lyso)phosphatidylcholine; LTPA: leisure-time physical activity; PSS: Perceived Stress Scale; RMSSD: Root Mean Square of the Successive R-R Differences; RP: reversed-phase.

The correlation analysis showed a positive association between these features and stress percentage (Fig. 3). Further, they correlated negatively with relaxation percentage and stress balance during sleep, and AAQW, which indicated a positive association with psychological flexibility. Also, we observed the negative correlations between these features and body fat, hsCRP, IL-1Ra, and adiponectin.

## Discussion

From the 8-week Elixir lifestyle intervention aimed to improve well-being, we profiled the fasting plasma metabolites of volunteers participated in the face-to-face intervention group and the control group for this substudy. Compared to controls, the intervention group had a more pronounced improvement in psychological well-being, indicated by higher relaxation and lower stress indices, as well as lower AAQW, referring to higher psychological flexibility^24^. Despite the lack of intervention effect on the metabolic profile, we showed the correlation between some phosphatidylcholines as well as another class of unidentified lipids and markers of stress, recovery, body composition, and inflammation.

Unlike a previous study in rats with chronic mild stress^25^, we found lower LPCs in the intervention group following the lifestyle intervention, with different correlations with physiological parameters based on the acyl chains. The stress relief, as indicated by a lower stress index, was positively associated with the suppressed LPCs in the intervention group: LPC(14:0), (16:1), and (20:4) (Cluster B, Fig. 2). LPC(14:0) and (16:1), in particular, also inversely correlated with increased relaxation and recovery indices, along with RMSSD which may imply activated parasympathetic nervous system to counterbalance stress^26^. Previously, LPC(14:0) and (16:1) have been associated with arterial stiffness^27^. Other suppressed PCs, including LPC(22:5), PC(16:0/16:1), and PC(18:0/20:3) (Cluster C, Fig. 2), correlated positively with the reduction in adiposity and inflammation, and inversely with increased relaxation index. The positive correlation between these PCs with the AAQW scores may also link to the improvement of weight-related psychological flexibility in the intervention group since the lower AAQW score reflects higher psychological flexibility. Noteworthy, these (L)PC indicative for stress and inversely for recovery contained medium-chain saturated fatty acids (SFA), particularly 16:0 and 18:0. Previously, phospholipids with increased saturation have been shown to enhance cellular stress signaling^28^. Since these (L)PC showed potential as biomarkers of stress, adiposity, and inflammation which, in this study, were reduced by the lifestyle intervention, this finding hence may support the hypothesis of shared mechanisms between psychological and physiological stress responses^8^.

In addition to the positive association between LPC(20:4) with stress index, the free fatty acid (20:4) form, has been previously suggested as a marker of depression and stress in human^18^ and rats^25^, respectively. LPC(22:5) in the intervention group was also associated with inflammation and adiposity. Interestingly, another PC containing polyunsaturated fatty acid (PUFA), PC(18:1/22:6), seemed elevated in the intervention group and correlated positively with the improvement of relaxation and recovery indices along with increased RMSSD. Hence, the PUFA that was bound in PC seemed to favour relaxation and recovery, whereas PUFA in LPC seemed to indicate stress, inflammation, adiposity, and inversely for relaxation and recovery. Although we cannot draw any causality in this study, this result might imply the possible role of PUFA-containing PC as a proxy of anti-inflammatory activity.

Another group that deserves attention is plasmalogens. The elevated plasmalogens PC(P-18:0/20:4) and PC(P-18:0/22:6) were inversely associated with adiposity indicators and AAQW, which related to better body composition and psychological flexibility. Also, LPC(P-18:0) was inversely associated with adiposity and inflammation markers hsCRP and IL-1Ra. The biological roles of plasmalogens as membrane component and anti-oxidant, also in the regulation of cell signalling, immune response, and cholesterol efflux, have been reviewed^29^. Further studies are thus required to segregate the structural and signalling functions of PC, containing either long-chain PUFA or medium-chain SFA, n-3 or n-6, based on their structure as LPCs or plasmalogens, in free or bound forms, along with relevant enzymes and metabolic pathways within a close framework of inflammation, adiposity, psychological and physiological stress responses.

Noteworthy, we did not observe any correlation between the two metabolites exhibiting the most significant difference in the metabolite profiling assay, namely retinol and epsilon-caprolactam with other parameters, except the negative relationship between epsilon-caprolactam with BMI and body fat percentage. One of the reasons behind the large effect size of retinol (Cohen’s D = −0.59, *p* = 0.002) could be the more substantial reduction of vitamin A intake in the intervention group than one in the control group, though it did not reach statistical significance (Supplementary Table S3). Therefore, we want to underline that in this free-living design, the volunteers were daily exposed to multiple stressors or other lifestyle factors which may alter the metabolic profile. In other words, the observed differences in the metabolic profiles could be attributable to other factors. The employed correlation analysis hence served as a verification step if the metabolite of interest was related to the psychological or physiological outcome measures that became the point of interest in this present study. The observation of several metabolites belonging to the same compound classes (e.g., PC) could also confirm the observed association. Using physical activity as an example, Table 1 showed that physical activity did not significantly change in either of the group, and the changes did not differ between the two groups. Among all 17 identified PC, only LPC(17:0) and (20:3) were shown to correlate with physical activity (Fig. 2), which may diminish the contribution of physical activity on the fasting plasma profile of the participants in this study. Nevertheless, our results here require more in-depth investigations to untangle the contribution of lifestyle factors to obtain the objective biomarker of stress, *e.g*., in a more controlled experimental design or various population groups with different lifestyle factors.

We also observed a more pronounced effect of time on the metabolic profile regardless of the intervention (Supplemental Fig. S1), which may refer to the improved markers of body composition (Table 1) and subjective indicators of psychological well-being both in the intervention and control groups (Table 2). Although the annotation could not continue due to lack of compounds’ MS/MS and reference spectra, particular lipids predominated the distinctive features for the time. The available MS/MS spectra from two features may imply a class of lipids with polar side-chain, and which were also associated with adiposity and stress indicators. This finding hence calls for targeted measurement of this specific class in future investigation on the metabolites associated with both physiological and psychological stress indicators. However, considering that these features increased similarly both in intervention and control groups, they may represent the contribution of other factors or potential confounders that may explain the improvement in the control group (Table 1), which may not be relevant to the study objective or be confounded by the selection bias. One example could be the weight-loss effort during the time interval by the control group who had the intention to lose weight^21^, as proven by the voluntary enrollment.

The strengths of this study include the novel application of a non-targeted metabolomics approach to assess the metabolic profile of participants showing psychological stress improvement. This application hence may unveil new inter-disciplinary research in eating behaviour, psychology, or other related fields. The multi-centre lifestyle intervention in working-aged adults with psychological stress and high risk to metabolic syndrome resembled the real-life setting with a higher likelihood of prolonged intervention and smooth translation to public health policy. The population was quite homogenous consisting of overweight-to-obese Finnish individuals, minimising the potential confounders from a genetic, culturally-linked dietary pattern, or other environmental factors, but may also limit the generalisability of findings to other populations.

We also admit other limitations in this study. The effect of different locations and seasons have been included in the statistical models as random effects, but we cannot exclude their total potential contribution to the metabolic profile. The inclusion of both men and women in this study may attenuate the observed intervention effects due to sex-based differences in stress indicators^30^ and hormonal cycle, especially for women. The given intervention did not contain education on a healthy diet or physical activity^19^, allowing the behavioural changes to what they believed would be beneficial for them, instead of a standardised measure of a specific factor. Other potential sources of bias include rather highly-educated Finns, mostly non-smokers, and inter-individual differences in response to stress. The subjective assessment of psychological well-being using PSS and AAQW questionnaires may introduce reporting bias. Thus, we also included the results from objectively-measured stress indicators derived from HRV measurement. This measurement, however, may also add other potential sources of confounders, including medication. Consequently, we excluded users of alpha- and beta-blockers in the statistical analyses that contained HRV-based parameters.

End measurement at the follow-up was both strength and limitation of the study. The interval between the end of the intervention (week 10) and follow-up (week 36) allowed the volunteers to maintain the newly-adopted lifestyle after the intervention, hence enabled detection of longer-term changes, including chronic inflammatory markers and stress markers. Based on that aim, we did not include potential acute stress markers or the possible effect of daily dietary intake in this particular substudy. However, the 26-week period from the end of the intervention to the follow-up might allow lifestyle modification in participants of both groups, which may attenuate the observed effect of the intervention.

In conclusion, we investigated a panel of fasting plasma metabolites that changed during a lifestyle intervention aimed to improve well-being, along with an improvement in psychological well-being and body composition. Despite the lack of intervention effect on the metabolite profile, we found correlations between various phospholipids, especially those belonging to the PC class, with body composition, stress, and recovery parameters, such as PC-containing PUFA with recovery and inversely with adiposity and inflammation. These findings hence allow us to generate hypothesis on specific metabolites and the potentially relevant metabolic processes which could be involved in the systemic response to stress relief. Our findings thus provide supportive evidence for further investigations aiming to unveil the role of the identified phospholipids and another class of unidentified lipids in the molecular interplay between psychological well-being and metabolic health, including other related factors such as body composition and inflammation. Future effort to illuminate the causal mechanisms linking psychological and metabolic health is thereby encouraged.

## Methods

### Study population and samples selection

339 overweight or obese individuals from Helsinki, Jyväskylä, and Kuopio with self-perceived and reported stress symptoms participated in the Elixir randomised-controlled lifestyle intervention study, in September 2012-June 2013 and January-October 2013^19^. They were 25–60 years of age, had self-reported BMI 27–34.9 kg/m^2^, psychological stress ≥3 points according to the General Health Questionnaire (GHQ-12)^31^, and access to an internet-connected computer. The participants were randomised to either one of the following four parallel groups for eight weeks: a web-guided Cognitive Behavioural Therapy (CBT)-based intervention, an Acceptance and Commitment Therapy (ACT) guided by a mobile application, an ACT-based intervention with face-to-face group meetings, or a control group^19^ (Fig. 1). The overall aim of the whole intervention was to evaluate the effect of these interventions on psychological flexibility, risk factors of metabolic syndrome, and general well-being^19^. The determination of sample size, randomisation procedure, and complete description of the outcome measures have been described in the protocol paper^19^.

The participants assigned to the face-to-face group meetings showed the most pronounced improvement of psychological flexibility during the intervention^23^. They were therefore included in this secondary analysis aimed to investigate the metabolic profile indicative for psychological well-being, along with the participants in the control group. After exclusion of non-compliant participants (attended less than four sessions of the face-to-face group meetings, drop-out due to personal reasons, or loss to follow-up), 64 and 60 volunteers from control and face-to-face intervention groups, respectively, completed the follow-up measurement (Fig. 1).

### Ethics

The protocol of Elixir study was approved by the ethics committee of the Central Finland Health Care District with the reference number 7U/2012 and was performed following the Declaration of Helsinki. The study was registered with ClinicalTrials.gov with identifier NCT01738256. All participants signed written informed consent during the laboratory examination at baseline. All data were handled anonymously.

### Study visits

The participants came to the study centres after an overnight fast at baseline (week 0), after the intervention (week 10), and follow-up (week 36), for venous blood sampling and measurement of body composition (body weight, height, body fat percentage, and waist circumference)^19^. Inflammation markers hsCRP, IL-1Ra, and high molecular weight adiponectin (HMW-adiponectin) were measured in the serum samples^19^. Metabolic profiling was performed on plasma samples obtained at the baseline (week 0) and follow-up (week 36) measurement (Fig. 1), comprising 248 samples for metabolomics analysis altogether. Plasma samples were stored at −80 °C until the laboratory procedure.

### LC-MS analysis

We used the in-house developed web application, Wranglr (https://github.com/antonvsdata/wranglr) to automate samples recoding, randomisation, and worklists generation for LC-MS experiments. As the input, Wranglr takes a spreadsheet containing necessary information about the samples such as sample identifier and study group. The output is worklists for LC-MS software containing quality control (QC) and autoMS/MS samples along with a processed spreadsheet containing sample information, injection order, plate positions, data file paths, and other relevant information. Wranglr is implemented in R 3.4.0^32^ using the Shiny package version 1.0.3^33^.

100 µl sample that has been properly thawed on ice was mixed with 400 µl of LC-MS-grade acetonitrile to obtain protein-free filtrate, while a small part of each sample was pooled down as a QC sample, as previously performed^34^. Liquid chromatography platform consists of 1290 Infinity Binary UPLC system (Agilent Technologies, Santa Clara, CA, USA) using both reverse-phase (RP, Zorbax Eclipse XDB-C18, particle size 1.8 µm, dimensions of 2.1 × 100 mm, Agilent Technologies, USA) and hydrophilic interaction chromatography (HILIC, Acquity UPLC® BEH Amide 1.7 µm, 2.1 × 100 mm, Waters, Ireland) columns with previously established protocol^35^. The coupling mass spectrometry was a 6540 UHD Accurate-Mass Q-TOF LC/MS (Agilent Technologies, Santa Clara, CA, USA) employing both positive and negative electrospray ionisation (ESI) modes^36^. Four precursor ions with the highest abundance were selected for MS/MS fragmentation with collision energies 10, 20, and 40 V. The low-abundance signals of interest which were not selected for fragmentation underwent targeted MS/MS analysis as described in the **Supplementary Method**. Due to a technical event, eight samples were not included in the RP positive mode, thereby the metabolic features from those eight samples were only supported by measurement in HILIC (both positive and negative modes) and RP negative mode.

### Initial peak-picking and alignment

The peak-picking procedure was initially performed using “Find by Molecular Feature” algorithm in Agilent MassHunter Qualitative Analysis B.07.00 (Agilent Technologies). Centroid spectra peaks higher than 400 counts were subjected against ion restriction: negative ions [M-H]^−^, [M + Cl]^−^, and [M + HCOO]^−^, and positive ions [M + H]^+^ and [M + Na]^+^. The data (.cef) files were then aligned and combined into a reference (.cef) file against which the original raw data was reanalysed in Mass Profiler Professional (MPP) software version 2.2 (Agilent Technologies). For this recursive analysis, we restricted the compounds with a maximum mass of 1000 Da and minimum absolute abundance of 5000 counts in negative modes or 8000 counts in positive ones. The tolerance for compound mass was 15 ppm ± 2 mDa, retention time ± 0.15 min, and symmetric expansion value ± 10 ppm for chromatograms. The output was re-exported to MPP for peak alignment and data cleanup. Subsequent filtering included only features that were present in at least 50% of samples in at least one study group. As the results, we got 2372 and 504 features from HILIC, and 3274 and 1334 features from RP, in positive and negative mode, respectively. The combined data matrix, which consisted of 7484 features from 248 samples with respective raw peak areas henceforth underwent data pre-processing.

### Data preprocessing: quality control and drift correction of metabolomics data

Before statistical analysis, signals that were present in less than 80% of the samples in all study groups and with detection rate less than 70% of the pooled QC samples were excluded. Signal quality was measured using error metrics defined previously^37^. The remaining signals were corrected for the drift pattern caused by the long LC-MS run. Regularised cubic spline regression was fit separately for each signal on the QC samples^38^. The smoothing parameter was chosen from an interval between 0.5 and 1.5 using leave-one-out cross-validation to prevent overfitting. The performance of the drift correction was assessed using robust non-parametric estimates of the relative standard deviation of QC samples (RSD*) and D-ratio*^37^ as quality metrics. Drift correction was only applied if the value of both quality metrics decreased, indicating enhanced quality. Otherwise, the original signal was retained.

After the drift correction, QC samples and low-quality signals were also removed before imputation and normalisation to prevent them from biasing the procedures. Signals were kept if their RSD* was <20% and their D-ratio < 40%, or if all of their classic RSD, RSD* and basic D-ratio < 10%. The missing values in the high-quality signals were imputed using random forest imputation implemented in the R package *missForest* version 1.4^39^. The out-of-bag error estimates were <0.1 for all the modes, indicating high-quality imputation. Following this procedure, we retained 1020 and 267 metabolic features from HILIC, and 1073 and 440 features from RP, in positive and negative mode, respectively. Signals were hereafter normalised using inverse-rank normalisation to approximate a normal distribution. The normalised data of each mode were then combined to a matrix of total 2800 features for statistical analysis.

### Other measurements

Because retinol had *p* < 0.05 for interaction between time and intervention, we hence checked if vitamin A intake also differed between the groups. Vitamin A intake was assessed based on a 48-hour dietary recall, including the portion size and preparation methods, performed by trained nutritionists. Alcohol consumption during the past six months was estimated using the Finnish version of Alcohol Use Disorders Identification Test-Consumption (AUDIT-C) questionnaire^40^. The LTPA was assessed using a questionnaire covering leisure-time and commuting activity and presented as metabolic equivalent (MET) multiplied by duration in hours (h)^19,41,42^. The sleep quality was based on a 7d-sleep diary^43^, with 1 indicating the worst and 5 indicating the best quality. The sleep efficiency was obtained based on an actigraphy measurement for one week, by dividing total sleep duration by the total time in bed, in percent^19^.

The assessment of psychological well-being employed questionnaire-based subjective indicators^19^. The measurement of stress used a 14-item PSS^44^ questionnaire to let the participants themselves indicate how stressful they had perceived their life during the previous month. A higher PSS score indicates a higher degree of perceiving one’s life stressful. Additionally, psychological flexibility was measured using Acceptance and Action Questionnaire for Weight-related difficulties (AAQW)^24^, with a lower score indicating higher psychological flexibility.

The objective assessment of stress and relaxation relied on a wearable HRV-monitoring device (Firstbeat Technologies, Jyväskylä, Finland). The participants were instructed to wear the device for three consecutive days and nights, consisting of two weekdays and one weekend day. Stress, recovery, and relaxation indices, stress and relaxation percentages, and stress balance during sleep were calculated using Firstbeat patented algorithms^45^. In brief, the recovery index estimated one’s recovery within 4 hours during sleep time, starting 30 minutes after going to bed. Stress and relaxation indices give absolute value characterising the magnitude of stress and relaxation, respectively. Conversely, stress and relaxation percentage calculate the percentage of stress and relaxation, respectively, within the measurement period. Stress balance during sleep gives a proportion between the time of stress and relaxation during sleep time (based on the description of the variables from Firstbeat Technologies). Based on the recorded HRV, the device also directly computed the RMSSD, which indicate the mean changes of the interval between two subsequent beats^46^. The average RMSSD was derived by calculating the average of RMSSD for each 5-minute interval during sleeping time.

### Statistical analysis for metabolomics data

A linear mixed model was fit separately for each metabolite to spot the differential metabolites using R packages *lme4*^47^ and *lmerTest*^48^. The effects of the intervention and time, along with their interaction, were modelled as fixed effects, with the interaction between intervention and time as the main result. The interaction term reflects different change during the intervention between the control and intervention group. Random effects included volunteers nested inside centres and the effect of season, crossed with the previous effects. All random effects were modeled as random intercepts, tested using the likelihood ratio test. The significance of the regression coefficients was tested using a t-test with Satterthwaite’s approximation for degrees of freedom. Confidence intervals were constructed using parametric bootstrapping percentile intervals with 1000 simulations. Benjamini-Hochberg false discovery rate (FDR) was performed for multiple testing correction on the unannotated 2800 features (Supplementary Table S1). Feature screening was then performed based on the unadjusted *p*, as we mainly use the *p*-value to rank metabolites for potential discoveries and hypothesis generation, not to prove statistically significant effects. All statistical analyses for metabolite signals were performed using R version 3.4.2^32^.

### Identification of compounds

The screening of features for the interaction between time and intervention was performed with the following criteria: unadjusted *p* < 0.05, mass < 1000 Da, and retention time > 0.5 min. After filtering, 104 features were subjected to the identification based on reporting guidelines from Metabolomics Standard Initiative^49^. Compounds with level I matched references in the in-house library built by running commercial standard in the same instrument and experimental condition. Level II includes compounds with matching mass, ion charge, and spectra of fragmented ions from online databases, such as METLIN^50^, the Human Metabolome Database^51^ (HMDB) version 3.6, or LIPID MAPS^52^. Identification of phospholipids was performed based on ion fragmentation pattern in the positive mode^53^, while the acyl-chain was determined based on the formate adduct of the corresponding compounds in the negative mode^54,55^. Level III includes unknown compounds with the prediction of compound classes or functional groups based on the MS/MS fragments. Level IV includes all unidentified metabolites. Eventually, 27 differential metabolites (7 from HILIC positive, 7 from RP negative, and 13 from RP positive), with their respective MS/MS fragments, were reported in Supplementary Table S1. The annotation step for the features differential for time was done with similar manner and following criteria: FDR < 0.05, RT > 1 min.

### Pathway analysis

The identified peaks differential for the interaction between time and intervention with level I and II identification were included in the “Pathway Analysis” module in MetaboAnalyst 4.0^56^, using their HMDB ID as the identifiers (Supplementary Table S4), except carnitine (11:1) and epsilon-caprolactam with lacking and unrecognized HMDB IDs, respectively. The analyses were then performed for the remaining 20 metabolites with *Homo sapiens* KEGG as the reference library and all compounds in the selected pathways as the reference metabolome. The over-representation analysis was performed using the Fisher exact test and the pathway topology using relative-betweenness centrality.

### Statistical analyses for other parameters

Due to the possible effects of medications containing alpha- and beta-blockers on heart rate, we excluded the volunteers using such medicines in all analyses using HRV-based parameters. Finally, 53 and 58 volunteers from the intervention and control groups, respectively, were included in analyses concerning HRV-based parameters (Fig. 1). After this exclusion, the data from both intervention and control groups at both baseline and follow-up time points were merged and included for the Pearson correlation analysis between the identified metabolites and the psychological and physiological parameters. The correlation patterns were visualised in a heatmap, where the metabolites, psychological, and physiological parameters were ordered using hierarchical clustering with Euclidean distance and Ward’s hierarchical clustering criterion (ward.D2)^57^. The effect sizes were calculated using Cohen’s D, derived by subtracting the mean difference in change (dI-dC) in the intervention (subscript I) and control (subscript C) groups then dividing it with the mean of the standard deviation of the changes in both groups.

$${\rm{Cohen}}{\rm{\mbox{'}}}{\rm{s}}\,{\rm{d}}=\frac{{\bar{{d}}}_{{I}}-{\bar{{d}}}_{{C}}}{1/2({s}{{d}}_{{dI}}+{s}{{d}}_{{dC}})};\,({{d}}_{{x}}={{y}}_{{xW}36}-{{y}}_{{xW}0});\,{\rm{x}}={\rm{I}}\,{\rm{or}}\,{\rm{C}}$$

where: ${\bar{{d}}}_{{I}}$ and ${\bar{{d}}}_{{C}}$ are the averages of the metabolite changes between the follow-up measurement (week 36) and baseline (week 0) in the intervention (I) group and the control (C) group, respectively. An absolute effect size of <0.25 were considered small, 0.25–0.5 medium, and >0.5 large^32,58^.

The differences between groups were analysed using Pearson’s Chi-Square for categorical variables. McNemar was used to comparing the changes within the dichotomous group proportion of alcohol users (users or non-users). A similar comparison was performed for smokers using the marginal homogeneity test for nominal data with more than two categories. The changes within (between week 0 and week 36) and between groups for continuous variables were compared using Wilcoxon signed-rank and Mann-Whitney U tests, respectively (IBM SPSS Statistics 25, International Business Machines Corporation). *P* < 0.05 was considered significant.

## Supplementary information

Supplementary materials SREP-19-25946B.
