# Supplementary material for: Plasma lipid profile associates with the improvement of psychological well-being in individuals with perceived stress symptoms
Source: Sci Rep. 2020 Feb 7;10:2143. doi: 10.1038/s41598-020-59051-x (PMC7005736; doi:10.1038/s41598-020-59051-x)
Supplement: Supplementary file 1 — Supplementary materials SREP-19-25946B. [file 41598_2020_59051_MOESM1_ESM.docx]

Plasma lipid profile associates with the improvement of psychological well-being in individuals with perceived stress symptoms

Stefania Noerman^1^, Anton Klåvus^1^, Elina Järvelä-Reijonen^1^, Leila Karhunen^1^, Seppo Auriola^2,3^, Riitta Korpela^4^, Raimo Lappalainen^5^, Urho M Kujala^6^, Sampsa Puttonen^7^, Marjukka Kolehmainen^1,#,*^, Kati Hanhineva^1,3,#^

^1^Institute of Public Health and Clinical Nutrition, University of Eastern Finland, Kuopio, Finland, P.O. Box 1627, FI-70211 Kuopio, Finland

^2^School of Pharmacy, University of Eastern Finland, Kuopio, Finland

^3^LC-MS Metabolomics Center, Biocenter Kuopio, Kuopio, Finland

^4^Medical Faculty, Pharmacology, Medical Nutrition Physiology, University of Helsinki, P.O. Box 63, FI-00014 University of Helsinki, Helsinki, Finland;

^5^Department of Psychology, Faculty of Education and Psychology, University of Jyväskylä, PO Box 35, FI-40014 University of Jyväskylä, Finland

^6^Faculty of Sport and Health Sciences, University of Jyväskylä, P.O. Box 35, FI-40014 Jyväskylä, Finland

^7^Finnish Institute of Occupational Health, P.O. Box 40, FI-00251 Helsinki, Finland

#Both authors shared the last authorship

*Corresponding Author: Marjukka Kolehmainen, University of Eastern Finland, Institute of Public Health and Clinical Nutrition, P.O. Box 1627, 70211 Kuopio, Finland. Phone: +358-40-3553617, E-mail: marjukka.kolehmainen@uef.fi

## Supplementary Method

## Targeted LC-MS analysis

Some unknown PCs which showed significant correlation with psychological or physiological parameters but not abundant enough for proper identification were run through the targeted LC-MS analysis. Top 10 samples containing most abundant metabolite of interest were marked. Targeted analysis for specific ionic mass and retention time on RP negative mode was performed on at least one of the marked samples for each metabolite of interest.

The temperature of the column and the sample tray were 50 °C and 4 °C, respectively. Isolation width for targeted data acquisition was 1.3 amu to acquire over the m/z 40-1700. The data were collected in the centroid mode with an abundance threshold 1000. Fragmentation was performed to all interesting ions listed for targeted analysis. The collision energies for MS/MS analysis were -10 and -20 V in subsequent runs. All setting parameters were same as non-targeted LC-MS analysis, except for the following parameters: sample flow rate 0.4 ml/min, acquisition rate 4 spectra/sec for MS scan and 2 spectra/sec for MS/MS scan. Data were acquired with Agilent MassHunter Acquisition B.07.00 (Agilent Technologies).

**Supplementary Table S1**

List of nominally differential (*p*<0.05) metabolites which indicated the interaction between intervention groups and time, sorted based on *p*

| Metabolite^3)^ | Column^1)^ | Mode | Mass | RT^1)^ | Adduct | Level | Main m/z spectra (relative intensity) | Estimate | 95% CI^2)^ | | | | | Cohen's D | *P*^2)^ | FDR^2)^ |
| --- | --- | --- | --- | --- | --- | --- | --- | --- | --- | --- | --- | --- | --- | --- | --- | --- |
| Retinol | RP | + | 268.221 | 10.47 | [M+H]+ | II | 93.069 (100), 69.070 (83), 107.086 (55), 105.069 (51), 81.069 (49), 119.086 (44), 55.054 (33), 79.053 (32), 95.086 (32), 121.102 (31) | -0.36 | ( | -0.60 | - | -0.13 | ) | -0.59 | 0.002 | 0.999 |
| PC (18:0/20:3) | RP | - | 857.613 | 13.22 | [M-H]- | II | (+): 812.617 (100), 184.073 (60); (-): 305.248 (100), 293.264 (30) | -0.49 | ( | -0.79 | - | -0.17 | ) | -0.54 | 0.003 | 0.999 |
| LPC (17:0) | Hilic | + | 509.348 | 1.15 | [M+H]+ | II | (+): 104.106 (100), 184.073 (80), 510.358 (72), 86.096 (18); (-): 269.249 (100) | -0.62 | ( | -1.05 | - | -0.18 | ) | -0.50 | 0.006 | 0.999 |
| Epsilon-caprolactam | RP | + | 113.084 | 3.01 | [M+H]+ | II | 114.092 (100), 44.013 (82), 55.055 (49), 69.070 (30), 79.054 (27), 96.082 (19), 67.055 (18) | 0.68 | ( | 0.22 | - | 1.17 | ) | 0.47 | 0.007 | 0.999 |
| Unknown Rppos_593.154@9.35 | RP | + | 593.154 | 9.35 | [M+H]+ | IV | 577.133 (100), 193.052 (13), 232.980 (11), 594.236 (11) | 0.59 | ( | 0.15 | - | 1.00 | ) | 0.52 | 0.008 | 0.999 |
| PC (18:0/22:5) | RP | + | 835.608 | 12.94 | [M+H]+ | II | (+): 184.073 (100); (-): 329.247 (100), 283.264 (36) | -0.43 | ( | -0.78 | - | -0.10 | ) | -0.47 | 0.014 | 0.999 |
| LPC (22:5) | RP | + | 569.349 | 10.10 | [M+H]+ | II | (+): 570.355 (100), 104.107 (75), 184.073 (75), 552.348 (11); (-): 554.325 (100), 329.248 (34) | -0.35 | ( | -0.64 | - | -0.07 | ) | -0.47 | 0.015 | 0.999 |
| LPC(17:1) | Hilic | + | 507.334 | 1.17 | [2M+H]+ | II | (+): 184.072 (100), 520.339 (52), 496.340 (36), 104.108 (16), 86.097 (16); (-): 267.233 (100), 492.307 (44), 268.237 (15), 224.067 (15) | -0.47 | ( | -0.83 | - | -0.09 | ) | -0.44 | 0.016 | 0.999 |
| PC(P-18:0/22:6) | RP | + | 817.596 | 12.58 | [M+H]+ | II | (+): 818.604 (100), 184.073 (64); (-): 862.597 (100), 802.574 (62), 327.237 (10), 562.350 (7), 104.532 (6), 403.260 (3) | 0.42 | ( | 0.07 | - | 0.76 | ) | 0.43 | 0.017 | 0.999 |
| Sphingomyelin d32:2 | RP | - | 718.525 | 11.25 | [M-H]- | II | \| (+): 184.073 (100), 86.095 (15); (-): 657.495 (100), 717.513 (14), 658.494 (14), 559.896 (10), 483.290 (10) \| \| --- \| | -0.32 | ( | -0.58 | - | -0.06 | ) | -0.43 | 0.019 | 0.999 |
| LPC (18:2) | RP | - | 565.338 | 9.88 | [M-H]- | II | (+): 184.074 (100), 86.096 (37); (-): 279.232 (100), 504.308 (54), 44.998 (9) | -0.45 | ( | -0.80 | - | -0.08 | ) | -0.43 | 0.019 | 0.999 |
| Unknown Rppos_216.173@8.35 | RP | + | 216.173 | 8.35 | [M+Na]+ | IV | 98.982 (100), 95.083 (47), 221.064 (46), 71.087 (31), 123.116 (28), 65.036 (24), 154.976 (23) | -0.59 | ( | -1.13 | - | -0.11 | ) | -0.44 | 0.023 | 0.999 |
| Carnitine 11:1 | RP | + | 327.242 | 6.92 | [M+H]+ | II | 328.249 (100), 85.028 (41), 144.100 (9), 60.081 (6) | -0.34 | ( | -0.64 | - | -0.05 | ) | -0.42 | 0.025 | 0.999 |
| Alanine | Hilic | + | 89.048 | 5.49 | [M+H]+ | I | 44.049 (100), 44.997 (24) | 0.51 | ( | 0.08 | - | 0.96 | ) | 0.40 | 0.027 | 0.999 |
| PC (18:1/20:3) | RP | + | 809.595 | 12.57 | [M+H]+ | II | (+): 810.601 (100), 184.073 (55); (-): 305.248 (100), 281.249 (29) | -0.39 | ( | -0.75 | - | -0.05 | ) | -0.40 | 0.028 | 0.999 |
| PC (16:1/18:2) | RP | - | 801.551 | 11.96 | [M-H]- | II | (+): 184.074 (100); (-): 279.233 (100), 253.218 (31) | -0.45 | ( | -0.83 | - | -0.05 | ) | -0.40 | 0.028 | 0.999 |
| Unknown RPneg_925.600@13.22 | RP | - | 925.600 | 13.22 | [M-H]- | IV | 112.985 (100), 44.998 (18), 283.264 (7) | -0.39 | ( | -0.72 | - | -0.04 | ) | -0.40 | 0.029 | 0.999 |
| LPC (14:0) | RP | - | 513.306 | 9.64 | [M-H]- | II | (+): 184.074 (100), 399.823 (21); (-): 227.201 (100), 452.278 (48), 44.998 (12) | -0.40 | ( | -0.74 | - | -0.04 | ) | -0.40 | 0.030 | 0.999 |
| PC(P-18:0/20:4) | RP | - | 839.603 | 12.72 | [M-H]- | II | (+): 184.074 (100); (-): 303.234 (100), 44.998 (21), 329.247 (18), 492.345 (12), 403.257 (1) | 0.30 | ( | 0.04 | - | 0.58 | ) | 0.39 | 0.033 | 0.999 |
| PC (16:0/16:1) | RP | + | 731.549 | 12.09 | [M+H]+ | II | (+): 184.074 (100); (-): 253.218 (100), 255.232 (48), 281.248 (27), 227.200 (13), 44.998 (12) | -0.35 | ( | -0.68 | - | -0.03 | ) | -0.36 | 0.033 | 0.999 |
| Phosphocholine | Hilic | + | 183.066 | 1.17 | [M+H]+ | I | 86.096 (100), 124.999 (82), 184.071 (17), 104.105 (12) | -0.38 | ( | -0.71 | - | -0.03 | ) | -0.38 | 0.036 | 0.999 |
| LPC (20:4) | Hilic | + | 543.332 | 1.09 | [M+H]+ | II | (+): 184.075 (100), 544.340 (8); (-): 303.232 (100), 224.071 (36) | -0.33 | ( | -0.63 | - | -0.02 | ) | -0.38 | 0.038 | 0.999 |
| PC(16:0/14:0) | RP | + | 705.532 | 12.00 | [M+H]+ | II | (+): 184.073 (100), 706.536 (59); (-): 690.508 (100), 227.201 (31), 272.989 (9), 255.235 (9), 750.526 (8) | -0.40 | ( | -0.78 | - | -0.03 | ) | -0.35 | 0.040 | 0.999 |
| LPC (16:1) | Hilic | + | 493.318 | 1.20 | [M+H]+ | II | (+): 494.324 (100), 184.074 (70), 476.313 (10); (-): 253.216 (100), 478.292 (47) | -0.32 | ( | -0.61 | - | -0.01 | ) | -0.37 | 0.044 | 0.999 |
| LPC (20:3) | RP | + | 545.350 | 10.16 | [M+H]+ | II | (+): 184.074 (100), 86.097 (20); (-): 530.324 (100), 305.248 (50), 531.324 (13), 480.306 (10), 44.998 (10) | -0.36 | ( | -0.73 | - | -0.02 | ) | -0.38 | 0.046 | 0.999 |
| Unknown RPPos_327.278@10.04 | RP | + | 327.278 | 10.04 | [M+H]+ | IV | 55.054 (100), 79.054 (61), 69.068 (57), 81.070 (56), 57.070 (42), 83.084 (33), 111.084 (27), 67.055 (27) | 0.37 | ( | -0.01 | - | 0.72 | ) | 0.39 | 0.048 | 0.999 |
| PC (18:1/22:6) | RP | + | 831.578 | 12.17 | [M+H]+ | II | (+): 832.585 (100), 184.072 (48); (-): 327.233 (100), 281.247 (41) | 0.31 | ( | -0.01 | - | 0.62 | ) | 0.36 | 0.049 | 0.999 |

1. HILIC: hydrophilic interaction column; RP: reverse-phase column; RT: retention time.
2. *P* values were determined using a linear mixed model with the interaction of intervention groups and time as the main effect, group and time as fixed effects, adjusted for seasonal and centre-based differences; positive estimates indicate increased levels in the intervention group or reduced levels in the control group, and vice versa; 95%CI: 95% confidence interval; FDR: Benjamini-Hochberg false discovery rate.
3. (L)PC: (lyso)phosphatidylcholine.

**Supplementary Table S2**

Metabolic pathways containing the identified changed metabolites in the intervention group

| Pathways | Total | Expected | Hits | Raw p | -LOG(p) | FDR | Impact |
| --- | --- | --- | --- | --- | --- | --- | --- |
| **Glycerophospholipid metabolism** | **39** | **0.081** | **3** | **3.85E-05** | **10.165** | **0.003** | **0.149** |
| Linoleic acid metabolism | 15 | 0.031 | 1 | 0.031 | 3.480 | 0.652 | 0 |
| Taurine and hypotaurine metabolism | 20 | 0.042 | 1 | 0.041 | 3.197 | 0.652 | 0.032 |
| Selenoamino acid metabolism | 22 | 0.046 | 1 | 0.045 | 3.103 | 0.652 | 0 |
| Retinol metabolism | 22 | 0.046 | 1 | 0.045 | 3.103 | 0.652 | 0.093 |
| Alanine, aspartate and glutamate metabolism | 24 | 0.050 | 1 | 0.049 | 3.018 | 0.652 | 0.057 |
| alpha-Linolenic acid metabolism | 29 | 0.060 | 1 | 0.059 | 2.833 | 0.673 | 0 |
| Cysteine and methionine metabolism | 56 | 0.116 | 1 | 0.111 | 2.197 | 1 | 0 |
| Arachidonic acid metabolism | 62 | 0.129 | 1 | 0.122 | 2.100 | 1 | 0 |
| Aminoacyl-tRNA biosynthesis | 75 | 0.156 | 1 | 0.147 | 1.921 | 1 | 0 |

The pathway analysis was performed in the MetaboAnalyst using the KEGG database for *Homo sapiens* as the reference library and Fisher exact test for the over-representation analysis. The pathway topology was performed using relative-betweenness centrality using all compounds in the selected pathways as the reference metabolome.

**Supplementary Table S3**

Participants' intake of vitamin A at baseline and after follow-up

|  |  | Groups | |  |
| --- | --- | --- | --- | --- |
| Vitamin A intake (µg/d) | | Intervention  (n=53) | Control  (n=58) | *p*  (I-C) |
|  | Baseline (week 0) | 1199 ± 2060 | 1013 ± 1131 | 0.410 |
|  | After follow-up (week 36) | 878 ± 723 | 954 ± 880 | 0.902 |
|  | Changes (week 36 - week 0) | -321 ± 2175 | -58 ± 1285 | 0.249 |
|  |  |  |  |  |
|  | p(week 36-week 0) | 0.141 | 0.790 |  |
|  |  |  |  |  |
|  | Cohen's D | -0.152 | | |

Presented values are mean ± SD.

*p* within groups and between groups were tested using Wilcoxon signed-rank and Mann-Whitney U tests, respectively.

Cohen's D indicates the differences between changes in intervention and control groups, divided by the mean of the standard deviation of the changes within both groups.

**Supplementary Table S4**

List of metabolites identified with level I and II confidence level included for the network analysis with their respective HMDB ID sorted based on the estimate

| Metabolite | HMDB ID | Estimate | *p* |
| --- | --- | --- | --- |
| LPC (17:0) | HMDB0012108 | -0.62 | 0.006 |
| PC (18:0/20:3) | HMDB0008046 | -0.49 | 0.003 |
| LPC (P-18:0) | HMDB0013122 | -0.47 | 0.016 |
| PC (16:1/18:2) | HMDB0008006 | -0.45 | 0.028 |
| LPC (18:2) | HMDB0010386 | -0.45 | 0.019 |
| PC (18:0/22:5) | HMDB0008055 | -0.43 | 0.014 |
| LPC (14:0) | HMDB0010379 | -0.40 | 0.030 |
| PC(16:0/14:0) | HMDB0007965 | -0.40 | 0.040 |
| PC (18:1/20:3) | HMDB0008079 | -0.39 | 0.028 |
| Phosphocholine | HMDB0001565 | -0.38 | 0.036 |
| LPC (20:3) | HMDB0010393 | -0.36 | 0.046 |
| Retinol | HMDB0000305 | -0.36 | 0.002 |
| LPC (22:5) | HMDB0010402 | -0.35 | 0.015 |
| PC (16:0/16:1) | HMDB0007969 | -0.35 | 0.033 |
| LPC (20:4) | HMDB0010395 | -0.33 | 0.038 |
| LPC (16:1) | HMDB0010383 | -0.32 | 0.044 |
| PC(P-18:0/20:4) | HMDB0011254 | 0.30 | 0.033 |
| PC (18:1/22:6) | HMDB0008090 | 0.31 | 0.049 |
| PC(P-18:0/22:6) | HMDB0011262 | 0.42 | 0.017 |
| Alanine | HMDB0000161 | 0.51 | 0.027 |
| Epsilon-caprolactam* | HMDB0062769 | 0.68 | 0.007 |

The estimate reflects how a metabolite would predict the mixed model, positive estimates indicate increased levels in the intervention group or reduced levels in the control group, and *vice versa*; *p* indicates the significance of the interaction between time and intervention as the fixed effects.

Regardless of the available HMDB ID, epsilon-caprolactam (marked with asterisk) was not recognised in the Pathway Analysis module in MetaboAnalyst.

**
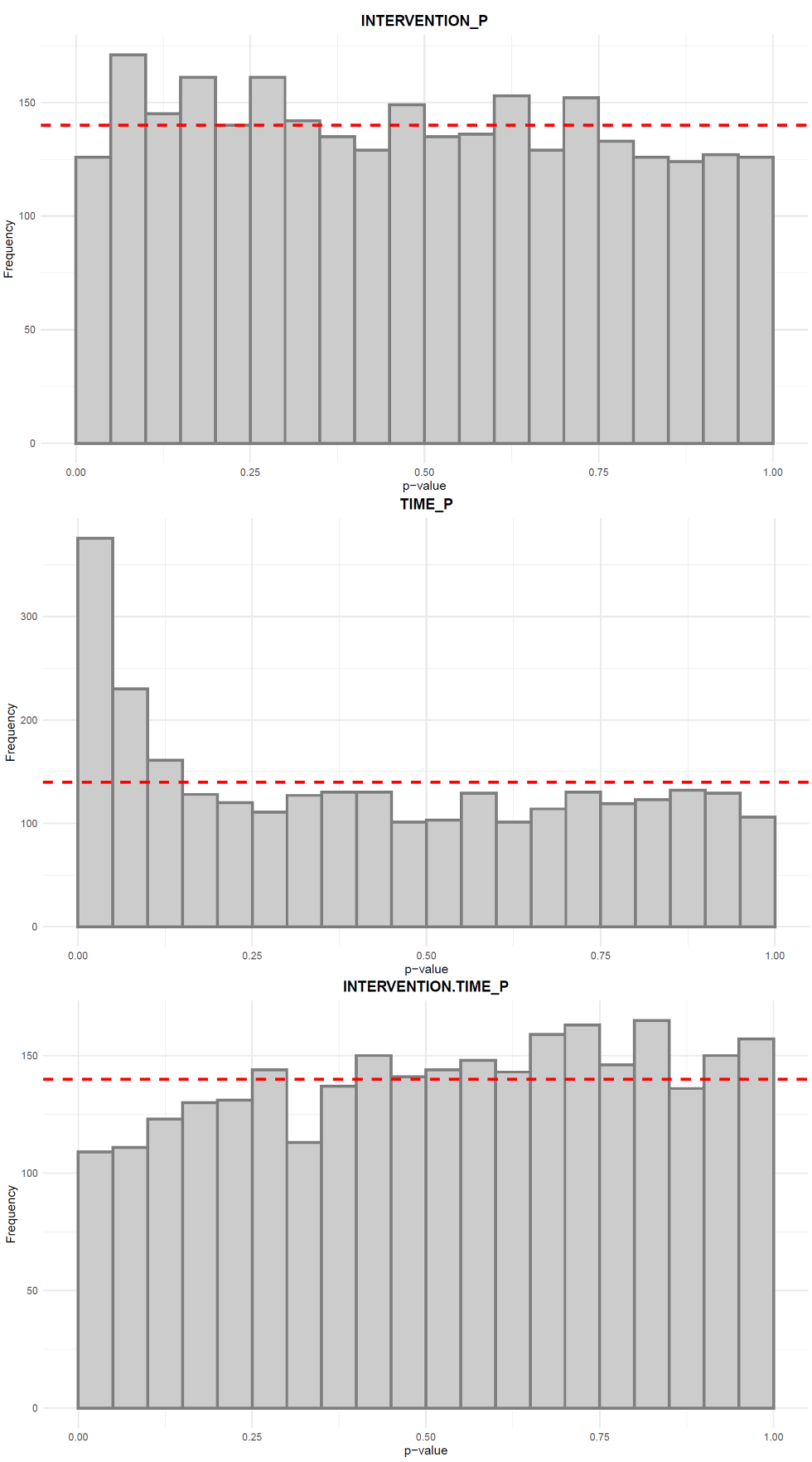
**

**Supplementary Figure S1**

Histogram of *p* distribution for the intervention (participation in face-to-face intervention or control group) and time (before the intervention, week 0 or after follow-up, week 36) and their interaction as fixed effects. The statistical model was a linear mixed effect model with volunteers nested inside centres and the effect of the season as random effects, crossed with the previous effects. The red lines indicate the expected frequency of obtaining such *p* at random.
